# Eco-Friendly and Complete Recycling of Waste Bamboo-Based Disposable Paper Cups for Value-Added Transparent Cellulose-Based Films and Paper Plastic Composites

**DOI:** 10.3390/polym14081589

**Published:** 2022-04-13

**Authors:** Peng Jia, Xiaoqian Ji, Bin Zheng, Chunyang Wang, Wenjie Hao, Wenjia Han, Jun Zhang, Guangmei Xia, Xingxiang Ji, Jinming Zhang

**Affiliations:** 1 State Key Laboratory of Biobased Material and Green Papermaking, Faculty of Light Industry, Qilu University of Technology (Shandong Academy of Sciences), Jinan 250353, China; skl_jiapeng@163.com (P.J.); jxq20021220@163.com (X.J.); skl_zhengbin@163.com (B.Z.); wcy15762143459@163.com (C.W.); hwj200506@163.com (W.H.); 2 Beijing National Laboratory for Molecular Sciences, CAS Key Laboratory of Engineering Plastics, Institute of Chemistry, Chinese Academy of Sciences (CAS), Beijing 100190, China; 2272037575a@sina.com (W.H.); jzhang@iccas.ac.cn (J.Z.)

**Keywords:** disposable cups, recycling, ionic liquid, cellulose, paper plastic composites

## Abstract

Disposable paper cups are widely used in daily life and most of them are landfilled or incinerated after use, resulting in a serious ecological hazard and significant waste of resources due to the usage of thin polyethylene (PE) as their inner coating. Hence, converting these common solid domestic wastes into high-value added materials is attractive and meaningful. In this study, transparent cellulose-based films were achieved from old bamboo-based disposable paper cups after pretreatment through using the room ionic liquid 1-allyl-3-methylimidazolium chloride (AmimCl) as solvent. The cellulose-based film with a dense texture demonstrated a relatively nice mechanical and UV-shielding performances, and its tensile strength was as high as 48 MPa, much higher than that of commercial polyethylene (PE, 12 MPa) film. Thus, the resultant cellulose-based film showed a great potential in the packaging field. Besides, the flexible paper plastic composites (PPC) were also fabricated from the rest thin PE coating with the stuck fibers, and it was found that PPC showed excellent mechanical property and hydrophobicity. Consequently, a feasible and eco-friendly process of recycling and reusing waste disposable paper cups was developed to achieve a complete utilization and valorization of waste disposable paper cups.

## 1. Introduction

To navigate away global catastrophic climate change, the target of carbon peak and carbon neutrality has been put forward worldwide [1,2,3], and renewable biomass resources have also dramatically aroused wide attention in the last decade [4,5,6,7]. As one of the oldest, cheapest, and most abundant biomass resources in nature, cellulose has been fabricated into a large quantity of regenerated cellulose products and cellulose derived materials, which exhibit extensive applications in material and energy fields [4,8,9]. However, the major sources of cellulose are still highly purified and dissolving-grade pulps such as wood and cotton pulp, leading to a high-cost of production which limits the regenerated cellulose materials development [10,11,12]. Fortunately, the low-cost cellulose-contained resources, such as agricultural wastes, forestry wastes, and waste cotton textiles [11,13,14,15,16], which are produced in large volumes and cultivated world widely, have become promising sources of cellulose.

Disposable paper cups (DPC) are usually made of thin internal polyethylene (PE, 5%) coating and high-grade virgin paper board (95%), which are cheap, clean, and convenient, so they are widely used in our work and life [17,18,19]. It has been found that approximately 2500 million paper cups are consumed in the United Kingdom, resulting in approximately 30,000 tons waste coffee cup every year, and the United States consumes more than 50,000 million paper cups annually [20]. In Australia, 720,510 trees are cut down to produce a billion coffee cups every year, leading to around 7000 tons of non-recyclable waste, and about 10,000 million disposable paper cups are bought in China each year [17,20]. As its name suggests, DPC has a short life and a grand of waste disposable paper cups (WDPC) are produced every year. Nevertheless, almost all of these waste disposable paper cups (WDPC) are discarded, and then incinerated in outdoors or dumped into landfill at the end of their service and fewer than one in 400 of WDPC is recycled and reused [20,21]. Therefore, using these low cost waste disposable paper cups to supplement or replace the traditional high-cost dissolving pulp and valorization of waste disposable paper cups to obtain cellulose-based materials is an attractive and significant aim.

Several investigations have been conducted to recycle and reuse WDPC [19,21,22,23,24], but few works have focused on preparing cellulose films from WDPC for the past few years [21], ince paper plastic laminates (PPL) are stuck tightly and separating them efficiently and economically is difficult. Adopting physical method, Jonathan et al. [22] incorporated the shredded disposable cups into polypropylene to achieve novel paper plastic composites (PPCs). Both tensile strength and young modulus of PPCs were increased by addition of PPL flake, demonstrating that the disposable paper cups can be recycled as reinforcement for the new polypropylene composites. The physical method is easy and efficient, but PPCs are eventually abandoned and pollute our environment. By adopting vermicomposting, Karthika et al. [19] investigated the degradation of waste paper cups via bacterial consortium and Eudrillus eugeinea, and their work demonstrated that the degradation period of paper cups was reduced obviously by using earth worm and microbes vermicomposting. But this process is complicated, time-consuming, and has low efficiency. In contrast, recycling and reusing the WDPC through chemical methods are considered as the most efficient process since high-valued products can be obtained. By using low cost WDPCs as raw materials, the graphene sheets and a high degree of substitution carboxymethylcellulose were successfully prepared, and these products showed potential applications in composites and functional materials [21,24]. Meanwhile, Nagarajan et al. [25] fabricated bio-eco based nanocellulose via citric acid hydrolysis recently. All of these chemical methods added high value to the WDPC. However, some of them show some disadvantages, including being environment unfriendly, inducing potential corrosion to the apparatus and so on. Meanwhile, up-cycling of PE coating and paper pulp within one process is rarely realized. Furthermore, few of them show the up-recycling of WDPC to new products with high-value.

Cellulose is a recalcitrant natural polymer and shows no solubility in common solvents. However, room ionic liquids (ILs) are new good solvents for cellulose and show many merits, including nonflammability, negligible vapor pressure, easy recyclability, good recoverability, and good ability to dissolve inorganic and organic substances [26]. Most importantly, ILs can dissolve cellulose effectively and have been popularly employed for the preparation of the cellulose-based materials, which have broad applications in medical, oil spill removal and many other fields [27,28,29,30,31]. In our previous studies, cellulose-based wastes, tea leaf [32], newspapers [11], agricultural wastes [13,14], and waste cotton textiles [12,16] were successfully converted into transparent regenerated cellulose-based films with a relatively good mechanical and thermal property through 1-allyl-3-methylimidazolium chloride (AmimCl) ionic liquid. In this work, by using the same ionic liquid AmimCl as solvent, WDPC can be dissolved directly and effectively after peeling off the inner PE coating without any pretreatment. Herein, the cellulose-based films and paper plastic composites (PPC) with good performances were fabricated, respectively. Additionally, the influence of coagulation baths (water and ethanol) on the changes of morphology and crystalline structure for the regenerated films were also studied systematically. Moreover, the structure and property of paper plastic composites (PPC) fabricated from the thin PE coating with the stuck fibers by hot press were also studied.

## 2. Materials and Methods

### 2.1. Materials and Reagents

The waste disposable paper cups (WDPC, 95% bamboo pulp and 5%PE coating) which were only used once and collected from households, were produced by Shanghai Yunrui Industrial Development Co., Ltd., Shanghai, China. Cotton pulps (CP), one kind of high-grade dissolving pulps, whose degree of polymerization (DP) was 530, was kindly supported by Shandong ICCAS-Henglian Biobased Materials Co., Ltd., Weifang, China. Commercial polyethylene (PE) film used as the reference was bought from the Jinan Hengyou New Material Technology Co., Ltd., Jinan, China. All raw materials were dried in vacuum at 90 °C for 48 h before the experiment. The solvent 1-allyl-3-methylimidazolium chloride (AmimCl) was also supported by Shandong ICCAS-Henglian Biobased Materials Co., Ltd., Weifang, China, and prepared according to our previous investigations [33]. Other chemical reagents such as ethanol (analytical grade) were also sold by Jinan Hengyou New Material Technology Co., Ltd., Jinan, China, and employed without further purification.

### 2.2. Peel off the Thin PE Coating from Waste Disposable Paper Cups

It is necessary to pretreat the waste disposable paper cups (WDPC) before the dissolution experiment. Initially, WDPC were cut and then immersed into the deionized water for 60 min at room temperature to loosen the adhesion between the PE coating and paper board. Secondly, the inner thin PE coating can be easily peeled off by hands from the brown paper board to achieve the pretreated waste disposable paper cups (named p-WDPC). Finally, p-WDPC were put into the oven to dry at 60 °C for several days and then ground by the household wall breaker for further use.

### 2.3. Valorization of Pretreated Waste Disposable Paper Cups

**Figure 1:**
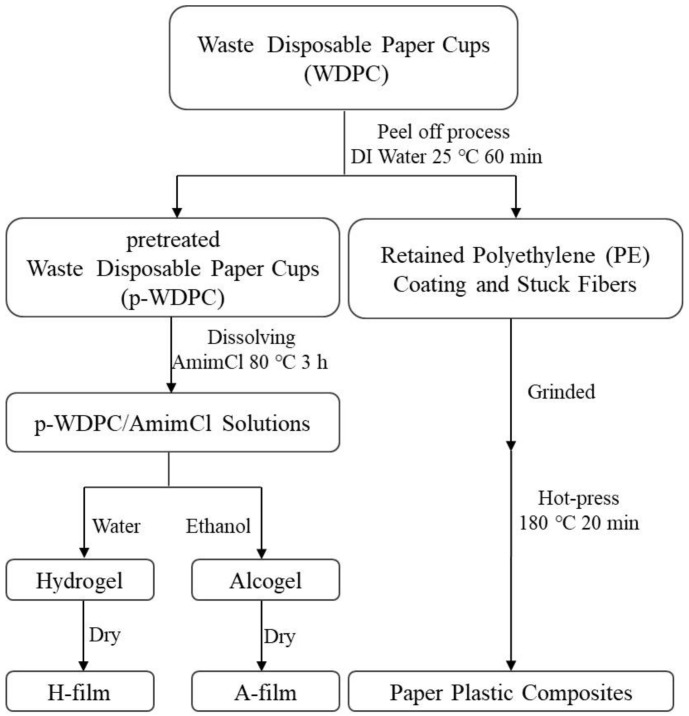
Preparation scheme of cellulose-based films (H-film and A-film) and paper plastic composites from waste disposable paper cups (WDPC).

**Figure 2:**
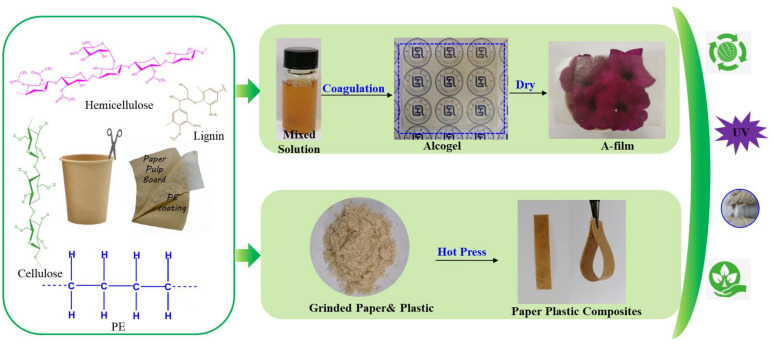
Process of converting waste disposable paper cups (WDPC) into cellulose-based films (H-film and A-film) and paper plastic composites (PPC) by AmimCl and hot press methods.

The dissolution process of waste disposable paper cups (WDPC) in AmimCl and the fabrication process of regenerated cellulose-based films (H-film and A-film) and paper plastic composites (PPC) are demonstrated in Figure 1. After peeling off the PE coating, the ground p-WDPC and AmimCl were poured into the three-necked flask. Then, the mixture was stirred vigorously and kept at 80 °C for 180 min to let the paper fibers dissolved completely. Finally, a homogeneous 2% p-WDPC/AmimCl solution was obtained. Subsequently, the solution was poured onto a flat plate and formed a 1000 μm thick layer with the spreader. Then, the glass plate covered with solution was soaked into the anti-solvent (ethanol or deionized water) of cellulose to prepare cellulose gels, where they were named alcogel and hydrogel, respectively. To get rid of the AmimCl completely, the gels needed to be washed by the corresponding anti-solvent at least five or six times until no Cl^−^ ions can be detected by the Ag^+^ test. The alcogel and hydrogel were dried by the Kessel paper dryer to prepare the regenerated films, which were named ‘A-film’ and ‘H-film’. Meanwhile, paper plastic composites (PPC) fabricated from the remained PE coating and pulp fibers stuck on PE could be obtained after hot press at 180 °C for 20 min. The process of transforming the waste disposable paper cups into cellulose-based films (A-film and H-film) and paper plastic composites by the ionic liquids solvent method and hot press is outlined in Figure 2.

For the purpose of comparison, the dissolution of high pure cotton pulp (CP) in AmimCl and the preparation of pure cellulose gel and film in a same method as depicted above were also conducted. Finally, C-gel and C-film were obtained from the high-grade cotton pulp (CP).

### 2.4. Characterization

#### 2.4.1. The Chemical Components in p-WDPC

GB/T 2677.8-1994, GB/T 10337-1989, GB/T 2677.10-1995, and nitric acid-ethanol method were adopted to detect the mass content of lignin, hemicellulose, and cellulose in p-WDPC.

#### 2.4.2. Degree of Polymerization (DP) of Waste Disposable Paper Cups Cellulose

By using cupriethylenediamine as a solvent, Ubbelodhe viscometry was employed to determine the degree of polymerization (DP) of cellulose in p-WDPC. The Standard Test Method for Intrinsic Viscosity of Cellulose (ASTM D795-13) was used as the reference. This experiment was conducted for at least three times and it was found that the DP was approximately 304.

#### 2.4.3. Polarized Optical Microscopy (POM) of p-WDPC/AmimCl Solution

It was necessary to use the POM to probe the dissolution ability of p-WDPC in AmimCl, so PM6000 polarizing microscope which was bought from Jiangnan Yongxin Optical Co., Ltd. (Nanjing, China) was adopted. A drop of p-WDPC/AmimCl solution was put onto the glass slide and a cover was also used. The micrographs of p-WDPC/AmimCl solution at 80 °C after 0 min, 30 min, 60 min and 180 min were recorded.

#### 2.4.4. Ultraviolet and Visible (UV-Vis) Spectroscopy of the Regenerated C-Film, H-Film and A-Film

The transparency and ultraviolet shielding performance of the regenerated C-film, H-film and A-film were characterized by Shimadzu’s UV 2600 ultraviolet spectrophotometer (Shimadzu, Japan), where the transmission mode was adopted.

#### 2.4.5. Mechanical Property of PE Film, H-Film, A-Film and PPC

The mechanical property of the regenerated H-film and A-film, commercial PE film and the PPC composites were characterized by the TA.XT Plus C texture analyzer which was produced by StableMicroSystem of Surrey in UK, where the drawing speed was 4.8 mm min^−1^. The specimens with 1 cm width and 5 cm length were employed and 2 cm of gauge length was kept in this work. It was necessary to test at least five specimens for each sample and the average value was displayed. Mechanical tests of the films were conducted according to the ASTM D-882 standard.

#### 2.4.6. Morphology of the WDPC, p-WDPC, PPC, H-Film and A-Film

The scanning electron microscopy (SEM) images of the WDPC, p-WDPC, PPC, H-film and A-film were obtained from the EM-30 Plus microscope which was purchased from the COXEM in Daejeon, Korea. In order to observe the cross-sections of PPC, H-film and A-film, the samples needed to be quenched in the liquid nitrogen. A thin layer of platinum was coated on all samples before observation.

#### 2.4.7. Fourier Transform Infrared (FTIR) Spectra of the WDPC, p-WDPC, Commercial PE Film, PPC, H-Film and A-Film

Fourier transform infrared (FTIR) spectra of the WDPC, p-WDPC, commercial PE film, PPC, H-film, and A-film was recorded by the ALPHA Fourier transform infrared spectrometer purchased from the Bruker in Karlsruhe, Germany, where attenuated total reflection (ATR) mode, 32 scans and 4 cm^−1^ of resolution were adopted for all samples.

#### 2.4.8. Wide-Angle X-ray Diffraction (WAXD) of the WDPC, p-WDPC, Commercial PE Film, PPC, H-Film and A-Film

The wide-angle X-ray diffraction (WAXD) patterns of the WDPC, p-WDPC, PE film, PPC, H-film and A-film was recorded by the D8 ADVANCE X-ray diffractometer bought from Bruker in Germany. 40 mA, 40 kV and CuKa Radiation (λ = 1.5406 Å) was used in this work. Meanwhile, the scan speed was set at 8°/min and patterns ranging from 5° to 50° (2θ) were displayed.

#### 2.4.9. Hydrophilicity of Commercial PE Film, PPC, C-Film, H-Film and A-Film

Hydrophilicity of commercial PE film, PPC, C-film, H-film and A-film was judged by their water contact angle, where Dataphysics OCA 50 bought from Filderstadt in Germany was used. For each film, five spots were tested and the average value was exhibited.

#### 2.4.10. Thermogravimetric Analysis (TGA) of the WDPC, p-WDPC, Commercial PE Film, PPC, H-Film and A-Film

The thermal performances of the WDPC, p-WDPC, commercial PE film, PPC, H-film and A-film were measured by employing the TA Q50 thermogravimetric analyzer, which was purchased from New Castle, Delaware, USA. A ceramic pan with a precision balance was set inside the furnace. It is necessary to cut the films into small pieces and approximately 5 mg of samples was put into the crucible pot. The test was conducted from the room temperature to 800 °C and the heating rate was set at 10 °C/min under the inert nitrogen atmosphere.

## 3. Results and Discussion

### 3.1. Dissolution and Pretreatment of Waste Disposable Paper Cups

Waste disposable paper cups (WDPC) were pretreated by the following processes and the state of the WDPC were depicted in Figure S1a–d. In order to peel off the inner PE coating, WDPC were firstly cut and then soaked in the deionized water for 1 h at room temperature (Figure S1a,c). After removing the thin PE, the pretreated waste disposable paper cups (p-WDPC) and the rest paper plastic (RPP) were dried in 60 °C for several days and kept in vacuum (Figure S1b). Finally, the p-WDPC and RPP were ground for further usage (Figure S1d). It can be seen that the p-WDPC became fluffy and RPP were homogenous, which benefited to the following dissolution and hot press processes, respectively.

**Figure 3:**
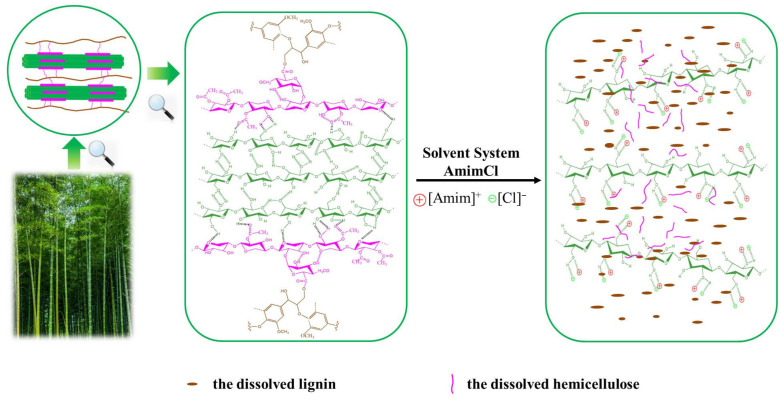
Dissolution mechanism of pretreated waste disposable paper cups in AmimCl solvent system.

The natural cellulose dissolved in ionic liquids was first announced by Rogers and his co-workers in 2002 [28]. It was found that chloride containing ILs appeared to be one of most effective solvents to dissolve cellulose. Moreover, it was also reported that lignocellulose can be dissolved in ionic liquids and AmimCl displayed excellent capacity for dissolving lignocellulose [28,34,35]. As demonstrated in Figure S2a–d, POM was employed to detect the detailed dissolution states of the p-WDPC/AmimCl. It can be noticed that p-WDPC contain copious microfibers, whose diameters ranged from 5 to 55 μm (Figure S2a). As time went by, the quantity of un-dissolved fibers decreased (Figure S2b,c) and nearly all of the fibers disappeared completely in AmimCl at around 80 °C after 180 min (Figure S2d), meaning that AmimCl realized the efficient dissolution of p-WDPC. The bamboo pulp is composed of 69.68% cellulose, 13.74% hemicellulose, lignin 12.53% and other additives. Meanwhile, the cellulose dissolution mechanism in AmimCl has been reported and was also demonstrated in Figure 3, in which both anion and cation of AmimCl play important roles in the cellulose dissolution and the homogenous mixture was achieved by their synergistic effect [36,37]. However, the dissolution mechanism of lignocellulose in the AmimCl solvent system is not clear so far and needs to be further studied and discussed.

### 3.2. Transparency and Hydrophilicity of C-Film, H-Film and A-Film

**Figure 4:**
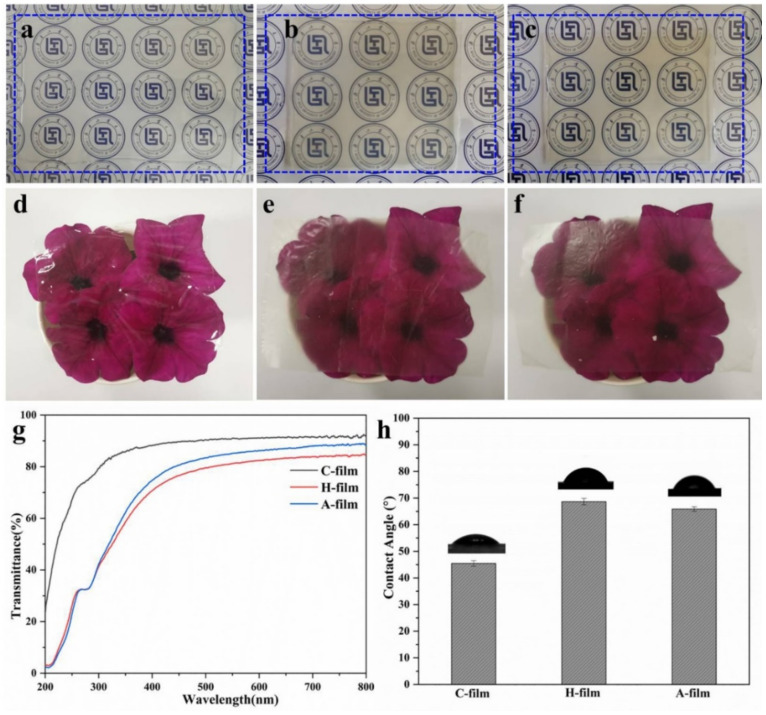
Optical pictures of the regenerated gels ((**a**) C-gel; (**b**) Hydrogel; (**c**) Alcogel) and films ((**d**) C-film; (**e**) H-film; (**f**) A-film); (**g**) UV-Vis spectra of C-film, H-film and A-film; (**h**) Water contact angles of the C-film, H-film, and A-film.

The transparency of materials affects their application and the optical pictures of cellulose-based gels and films are exhibited in Figure 4a–f. It can be concluded that both the cellulose-based gels (Figure 4a–c) and films (Figure 4d–f) display a relatively high transparency. However, the transparency of the C-gel and C-film prepared from cotton pulp, a high-cost and high-grade pure cellulose source, show the best transparency. The UV-vis transmittance quantifiably describes the difference in transparency (Figure 4g). Compared with the C-film, both the H-film and A-film possess a relatively low transmittance in visible region (400–800 nm), sinec lignin or other additives are contained in the regenerated H-film and A-film. However, the films prepared from the waste disposable paper cups still display good transparency (81.3% and 85.2% at 550 nm for H-film and A-film, respectively). It is worth noticing that A-film exhibits about 4% higher transmittance than that of H-film, indicating that the coagulation bath impacts significantly on the transparency of cellulose-based films. Fortunately, the H-film and A-film show natural UV-shielding property since bamboo-based pulp contains lignin, suggesting that H-film and A-film fabricated from natural bamboo-based disposable paper cups show great potential in ultraviolet shielding packaging fields. The water contact angles (WCA) of the regenerated cellulose-based films (C-film, H-film and A-film) are around 45.5°, 68.7°, and 65.9°, respectively (Figure 4h), implying that the regenerated C-film, H-film, and A-film display good wettability. It can be noticed the films (i.e., H-film and A-film) regenerated from WDPC have a bigger WCA, which is also attributed to the existence of hydrophobic lignin or other additives. In other words, compared with the pure cellulose films (C-film), the regenerated films made from bamboo-based pulp disposable paper cups possess good ultraviolet shielding and hydrophobicity, demonstrating their superiority in wrapping and packaging fields.

### 3.3. Structure, Thermal Degradation and Mechanical Property

**Figure 5:**
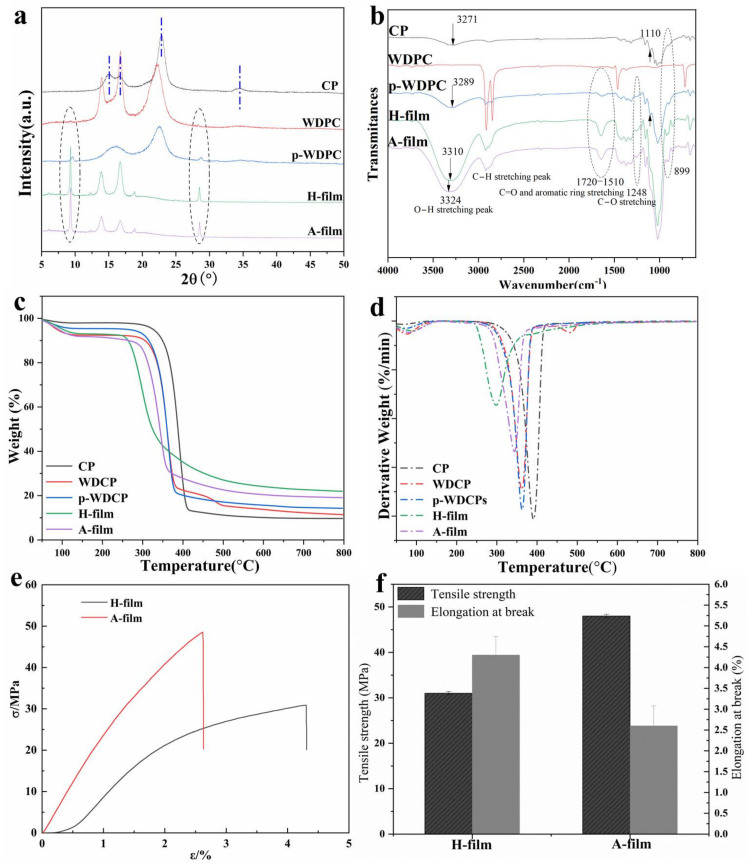
(**a**) XRD, (**b**) FTIR curves, (**c**) TG and (**d**) DTG of CP, WDPC, p-WDPC, H-film and A-film; Stress-strain curves (**e**), tensile strength and elongation at break (**f**) of the H-film and A-film.

Generally, the phase transformation of cellulose occurs after the cellulose dissolution, coagulation and drying processes. To investigate the changes of the crystalline phase, the X-ray diffraction patterns of CP, PE film, WDPC (the PE coating side), p-WDPC, H-film and A-film are drawn in Figure 5a and Figure S3. It is well known that the 2θ = 15.1°, 16.8°, 22.8° and 34.5°, corresponding to the (1–10), (110), (200) and (004), are crystal planes of cellulose I [11,38]. The CP, WDPC and p-WDPC exhibit well-defined peaks at the above positions, meaning that a typical cellulose I crystalline structure exists in the raw materials. However, the peeks at 2θ = 15.1° and 22.8° of WDPC shift to the lower degree since they are overlapped with those of PE coating (Figure S4). Furthermore, both WDPC and p-WDPC also show two minor peeks at 9.3° and 28.5°, which are also the typical peaks of PE coating. As presented, XRD patterns of regenerated-based films display prominent peeks at 9.3°, 13.8°, 21.6 and 23.8°, which are ascribed to the peaks of PE residue, implying that PE residue still retained in the p-WDPC, H-film and A-film. It is well known that waste paper cups are usually comprised of the internal thin PE coating and the outer paper pulp board. Since it is easy for the melt PE to permeate into paper board voids and then PE sticks to paper board tightly, separating the PE coating from the paper board completely is difficult, leading to the disposable paper cups can’t be effectively recycled or reused in industry. Therefore, decreasing the usage of disposable paper cups and developing environmentally friendly coating are meaningful and important. Beside cellulose changing from phase I to II after the dissolution, coagulation and drying processes, a decrease in intensity of the regenerated films (H-film and A-film) was also displayed, meaning that the crystallinity index of the products decreased sharply [11,13], so the peeks of PE residue are enhanced in H-film and A-film. Additionally, the XRD curves of H-film and A-film are similar with each other, suggesting that there is no obvious difference in crystalline structure of them.

FTIR can provide the component and structure information. Figure 5b and Figure S4 illustrate the spectra of CP, PE film, WDPC, p-WDPC and the regenerated films. The CP, p-WDPC and cellulose-based films show similar FTIR spectra with only minor differences, suggesting cellulose is the main composition of p-WDPC and no chemical reactions occur in this regeneration process [11]. However, the spectrum of WDPC (the PE coating side) was obviously different from that of the CP or p-WDPC, and it is similar with that of PE film, meaning that peeling off process can remove most of the PE coating from the surface of the bamboo-based paper board. Meanwhile, the O-H stretching peak ranging from 3200 to 3700 cm^−1^ is very sensitive to changes of cellulose crystalline structure [11]. As displayed in Figure 5b, the O-H stretching peak of CP and p-WDPC are located at around 3270 cm^−1^ and 3290 cm^−1^, respectively. However, the O-H stretching peak displays a blue shift to higher wavenumbers for the regenerated films (H-Film, 3310 cm^−1^ and A-Film, 3324 cm^−1^). Moreover, the peek at approximately 899 cm^−1^, which is weak in spectra of raw materials, is enhanced for the regenerated films (H-film and A-film), while the band at 1110 cm^−1^ which is significant for original CP and p-WDPC, disappears for the regenerated films, indicating the changes of phase and hydrogen-bonding after the dissolution, coagulation and drying processes, consistent with XRD data [11]. Additionally, compared with CP, p-WDPC, H-film and A-film show an obvious new widen band ranging from 1510 cm^−1^ to 1720 cm^−1^, which is attributed to the overlapped peak of hemicellulose (1740 cm^−1^, C=O stretching band) and lignin (1510 cm^−1^, aromatic ring stretching band). Meanwhile, the minor peak at 1248 cm^−1^ is also the peak of lignin [11,14,39]. Therefore, besides containing the main component cellulose, p-WDPC, the H-film and A-film also embody hemicellulose and lignin, which is in line with the analysis of the chemical compositions in p-WDPC.

It is well known that the thermal stability of materials is also very important for their application, so TGA was conducted to depict thermal performances of CP, WDPC, p-WDPC, PE film and the regenerated H-film and A-film. The curves of differential thermogram (DTG) and thermogram curves (TG) are both recorded, as exhibited in Figure 5c,d and Figure S5. Generally, the remained moisture is firstly removed and shows a minor peek at temperature below 200 °C. Cotton pulps (CP) demonstrates the highest temperature of maximum weight loss rates (T_max_, 395 °C) and onset decomposition temperature (T_onset_, 280 °C), while the T_max_ (360 °C) and T_onset_ (260 °C) of WDPC and p-WDPC are lower than those of CP, since the DP of CP (530) is bigger than that of p-WDPC (304). Meanwhile, the residue weights of WDPC and p-WDPC are bigger than that of CP, which may be ascribed to the lignin and other additives retained in the bamboo-based pulp. It is can be noticed that WDPC shows a minor peek at about 480 °C, ascribed to the T_max_ of the thin PE coating, which is the thermal feature of mechanically mixed composites. The commercial PE film possessed the T_max_ at 485 °C (Figure S5), indicating that the thin PE coating still displays similar thermal performance with that of commercial PE film. As presented, both the H-film and A-film degrade above 200 °C, attributed to decomposition of macromolecules chains [15]. Moreover, the T_onset_ and T_max_ of the regenerated H-film and A-film are smaller than that of p-WDPC, suggesting that the regenerated cellulose-based materials (H-film and A-film) possess low thermal stability, since the degree of cellulose polymerization and crystallinity decline after dissolving, coagulation and drying processes [15,40]. It is noteworthy that the T_max_ of H-film (300 °C) is about 40 °C lower than that of A-film (340 °C), due to the lower degree crystallinity of the H-film, meaning that the coagulation bath shows obvious influence on the thermal stability of the regenerated cellulose-based materials, which was also reported in our previous work [16].

The mechanical properties of membrane materials are important indexes for their application as the packaging materials. Figure 5c and Figure S6 demonstrate the typical stress-strain curves of the H-film, A-film, and PE film. The thickness of the cellulose-based films is around 18 μm (Table S1). Generally, the degree of polymerization (DP) of polymers has a direct effect on their mechanical property. As measured by Ubbelodhe viscometry, the DP of p-WDPC cellulose is about 304, indicating that the regenerated H-film and A-film processed good mechanical performances since the dissolution of cellulose in AmimCl is a mild dissolution process and cellulose DP decreases weakly under the mild conditions. Hence, the regenerated H-film and A-film display good tensile strengths ranging from 20 to 50 MPa (Figure 5e,f). Moreover, compared with the H-film (31 MPa, 4.3%), the A-film has lower elongation at break (2.6%) but higher tensile strength (48 MPa) due to the higher degree of crystallinity, indicating that mechanical performances of films obviously depend on their structure. It is worth noting that the H-film and A-film demonstrated higher tensile strength than that of commercial polyethylene films widely used in life (PE: 12 MPa, Figure S6), showing potential as packing and wrapping materials. It is well known that the elongation at break of the cellulose-based films is usually below 5% due to the rigid backbone structure of cellulose molecules, limiting their usage [13,14]. However, adding plasticizers can effectively improve the elongation at break of them [41]. Hence, these cellulose-based films obtained from waste paper cups exhibit huge possibility to supplement or replace PE or polypropylene (PP) films in packaging and coverage fields to follow sustainable development and tackle global climate change.

### 3.4. Morphology of Cellulose-Based Films

**Figure 6:**
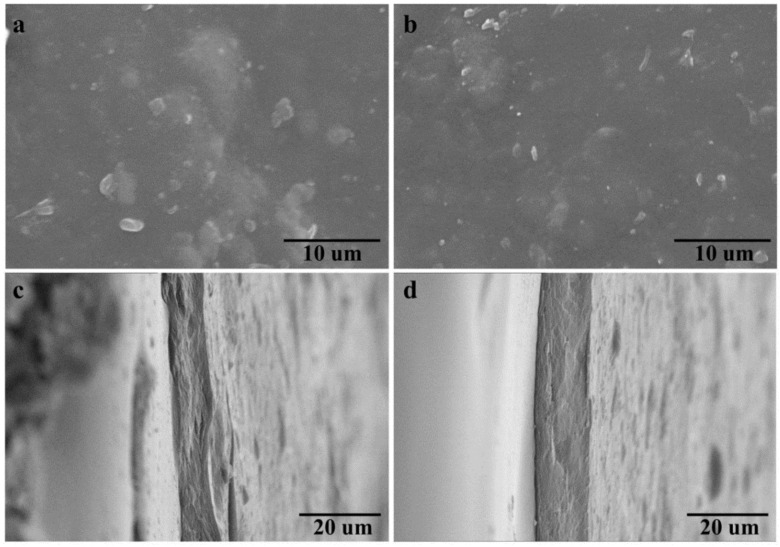
(**a**–**d**) SEM images of the regenerated H-film and A-film. (**a**,**b**) the surface of H-film and A-film; (**c**,**d**) the cross section of H-film and A-film.

To investigate the surface and cross section morphology of H-film and A-film, SEM micrographs were also output. Figure 6 shows that both H-film and A-film possess a relatively flat surface although PE residues, lignin and other impurities are contained in the films, due to the excellent solubility of AmimCl for lignocellulose. Meanwhile, both H-film and A-film show a dense inner texture, but the cross section of A-film is more homogeneous than that of the H-film, indicating that the structure of regenerated cellulose-based materials is significantly influenced by coagulation bath, which also may be another reason for the high tensile strength of the A-film.

### 3.5. Structures and Properties of Paper Plastic Composites (PPC)

**Figure 7:**
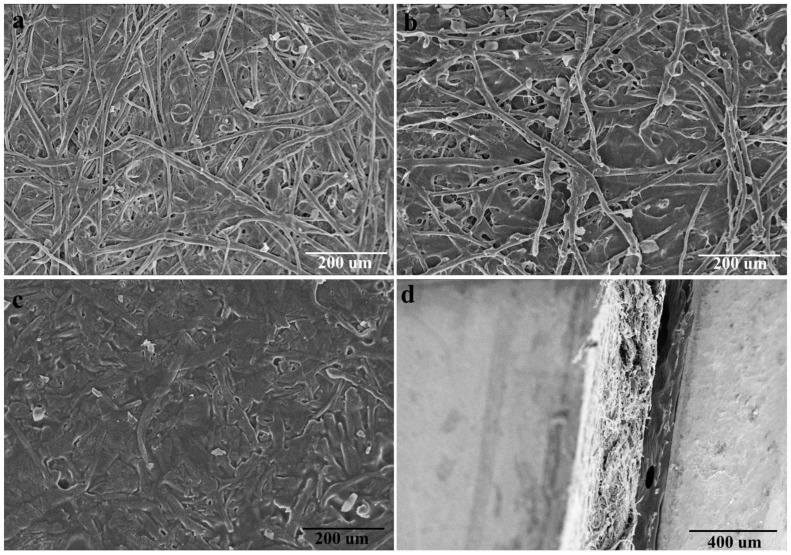
(**a**,**b**) The SEM images of waste disposable paper cups (WDPC) inner surface before and after peeling off the thin PE coating; (**c**,**d**) SEM images ((**c**), the surface and (**d**), the cross section) of the paper plastic composites (PPC).

To completely utilize the waste disposable paper cups via a simple and eco-friendly process, the remaining paper plastic (RPP) was fabricated into paper plastic composites (PPC) by hot press in this work. SEM images are recorded to examine the difference of waste disposable paper cups (WDPC) inner surface before and after peeling off the thin PE coating (Figure 7a,b). It can be seen that the outlines of bamboo fibers are still clearly although the paper board is coated with a waterproof thin PE. The prepared disposable paper cups display a relatively smooth and flat surface due to the PE coating. In contrast, the inner surface of WDPC becomes much coarser and three-dimensional structure of bamboo fibers appears more obviously after peeling off the PE coating. However, some PE residue is still retained in the network of the bamboo paper board, limiting the recycle and reuse of WDPC, since it is difficult to remove the PE from the paper board completely and efficiently. Therefore, complete utilization and valorization of waste disposable paper cups by a relatively simple, feasible, and green method in this work is attractive and meaningful. The surface and cross section pictures of PPC are illustrated in (Figure 7c,d). It can be concluded that the well-distributed PPC can be fabricated by melt pressing, where the melted PE and the intact fibers interact with each other and form a reinforced concrete structure, suggesting that PPC have good mechanical properties.

**Figure 8:**
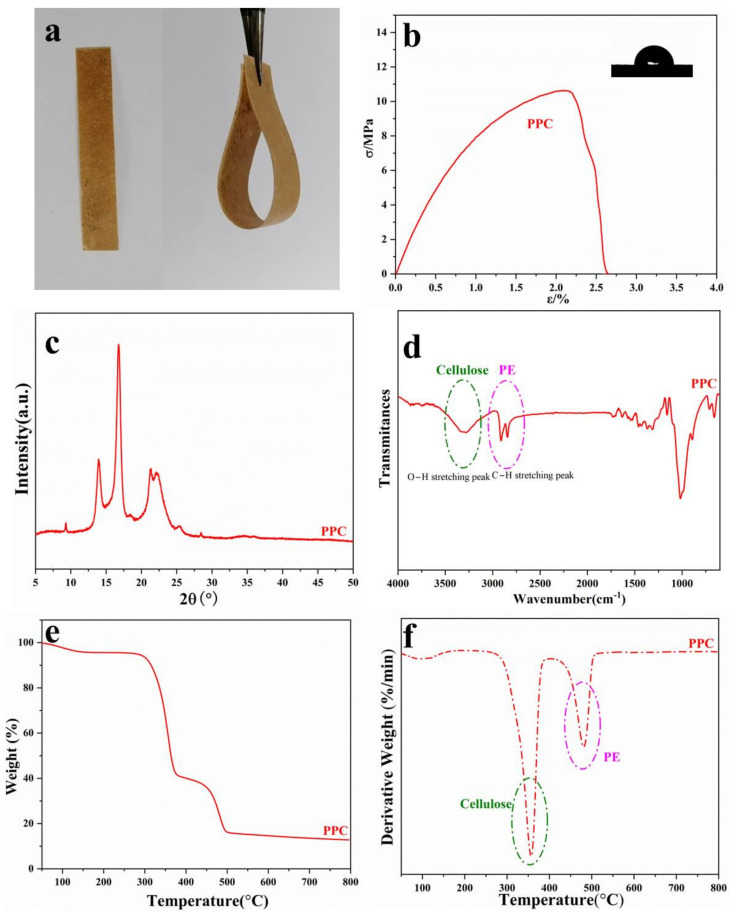
The digital picture (**a**), stress-strain curve (**b**), XRD (**c**), FTIR (**d**) and SEM ((**e**), the surface and (**f**), the cross section) of PPC.

To investigate the structure and property of the PPC, the digital picture, water contact angle, stress-strain curve, X-ray diffraction profile, FTIR spectrum, TG and DTG curve were recorded and are presented in Figure 8. PPC prepared from the thin PE with the stuck fibers were obtained after hot press at 180 °C for 20 min and Figure 8a exhibits the digital pictures of PPC. It can be seen that PPC are brown since lignin is contained bamboo pulp. Meanwhile, PPC possess good flexibility. Furthermore, the PPC have excellent mechanical property with the tensile strength of about 11 MPa (Figure 8b). Additionally, the water contact angle of PPC is 92.1°, bigger than that of commercial PE film (71.7°, Figure S7), displaying an attractive hydrophobicity, which can be a good supplement in packaging and express delivery industry. The main components of PPC are PE and cellulose, and therefore PPC show both the characteristics peeks of cellulose and PE, which can be seen from the XRD (Figure 8c) and FTIR data (Figure 8d). It can be noted that the PPC also display good thermal stability, and have two temperatures of maximum weight loss rate (T_max_: 360 °C and 480 °C), which are attributed to the bamboo fibers and original PE coating of WDPC [22], a general phenomenon of physical blending polymer materials (Figure 8e,f). It is worth speaking that PPC show more clear temperature of maximum weight loss rates of PE coating compared with that of WDPC, suggesting that the mass ratio of PE coating in PPC is bigger than that of WDPC, which is consistent with the fact. In short, PPC prepared from the rest PE coating of waste disposable paper cups still show homogeneous structure and possess good properties.

## 4. Conclusions

By using the low-cost waste disposable paper cups as source, the flexible paper plastic composites (PPC) and transparent regenerated cellulose-based films (H-film and A-film) were successfully fabricated through the ionic liquid (AmimCl) and hot press methods. There was a phase I to II transition after the cellulose dissolution, coagulation, and drying processes, which impacted the properties of the regenerated cellulose-based materials. The regenerated films showed a compact and transparent texture and possessed certain UV-shielding property, but their structure and property were impacted obviously by the coagulation bath. Moreover, the wettability and mechanical properties of the regenerated films were relatively good, and their tensile strengths ranged from 20 to 50 MPa, which is much higher than that of petroleum-based PE plastics (12 MPa) film. It was indicated that the waste disposable paper cups can become the supplement or substitute to high-cost and high-grade pulp as the source to fabricate cellulose-based films, which displayed huge possibility to replace polyolefin PP and PE films in packing fields to meet sustainable development goals and tackle global climate change. Generally, disposable paper cups (DPC) usually contain a large quantity of high-quality virgin cellulose paper board, which may be a good raw material to prepare cellulose products. However, recycling and reusing waste paper cups (WDPC) is difficult and studies on it were rare since the inner thin PE coating are bonded tightly with the cellulose paper board and separating them efficiently and economically is difficult. In order to completely utilize the WDPC, the flexible paper plastic composites (PPC) which had the features of paper and plastic were also prepared from the inner thin PE coating with the stuck fibers by simple hot press at 180 °C for 20 min. The tensile strength of PPC was approximately 11 MPa and the water contact angle was 92.1°, bigger than that of commercial PE film (71.7°), which can be a good supplement in packaging and transportation industry.

In conclusion, preparing high-valued cellulose-based films and paper plastic composites from the waste disposable paper cups by a simple and effective method can be realized in this work. Furthermore, the low-cost waste disposable paper cups can become the new supplement or substitute high-cost and high-grade pulp as a source of cellulose-based products which are good for the environment and for human use.

## Data Availability

The data presented in this study are available on request from the corresponding author.

## Supplementary Materials

The following supporting information can be downloaded at: https://www.mdpi.com/article/10.3390/polym14081589/s1. Figure S1: The photographs of (a) waste disposable paper cups (WDPC), (b) paper pulp board and remained paper plastic (RPP), (c) waste disposable paper cups soaked in deionized water and (d) shredded paper pulp board (p-WDPC) and ground RPP; Figure S2: POM micrographs (10 × 10) of p-WDPC/AmimCl solution at 80 °C after 0 min (a), 30 min (b), 60 min (c) and 180 min (d); Figure S3: The XRD profile of commercial PE film; Figure S4: The FTIR spectrum of commercial PE film; Figure S5: The (a) TG and (b) DTG curve of commercial PE film; Figure S6: The stress-strain curve of commercial PE film; Figure S7: The water contact angle (WCA) of commercial PE film; Table S1: The thicknesses of the C-film, H-film and A-film.
