# Supplementary material for: Eco-Friendly and Complete Recycling of Waste Bamboo-Based Disposable Paper Cups for Value-Added Transparent Cellulose-Based Films and Paper Plastic Composites
Source: Polymers (Basel). 2022 Apr 13;14(8):1589. doi: 10.3390/polym14081589 (PMC9028521; doi:10.3390/polym14081589)
Supplement: Supplementary file 1 [file polymers-14-01589-s001.zip › polymers-1674192-supplementary.pdf]

polymers MDPI

Supplementary Materials: Eco-Friendly and Complete Recycling of Waste Bamboo-Based Disposable Paper Cups for Value-Added Transparent Cellulose-Based Films and Paper Plastic Composites

Peng Jia, Xiaoqian Ji, Bin Zheng, Chunyang Wang, Wenjie Hao, Wenjia Han, Jun Zhang, Guangmei Xia, Xingxiang Ji and Jinming Zhang

Table S1. The thicknesses of the C-film, H-film and A-film.

Sample	Thickness (μm)
C-film	18
H-film	17
A-film	19

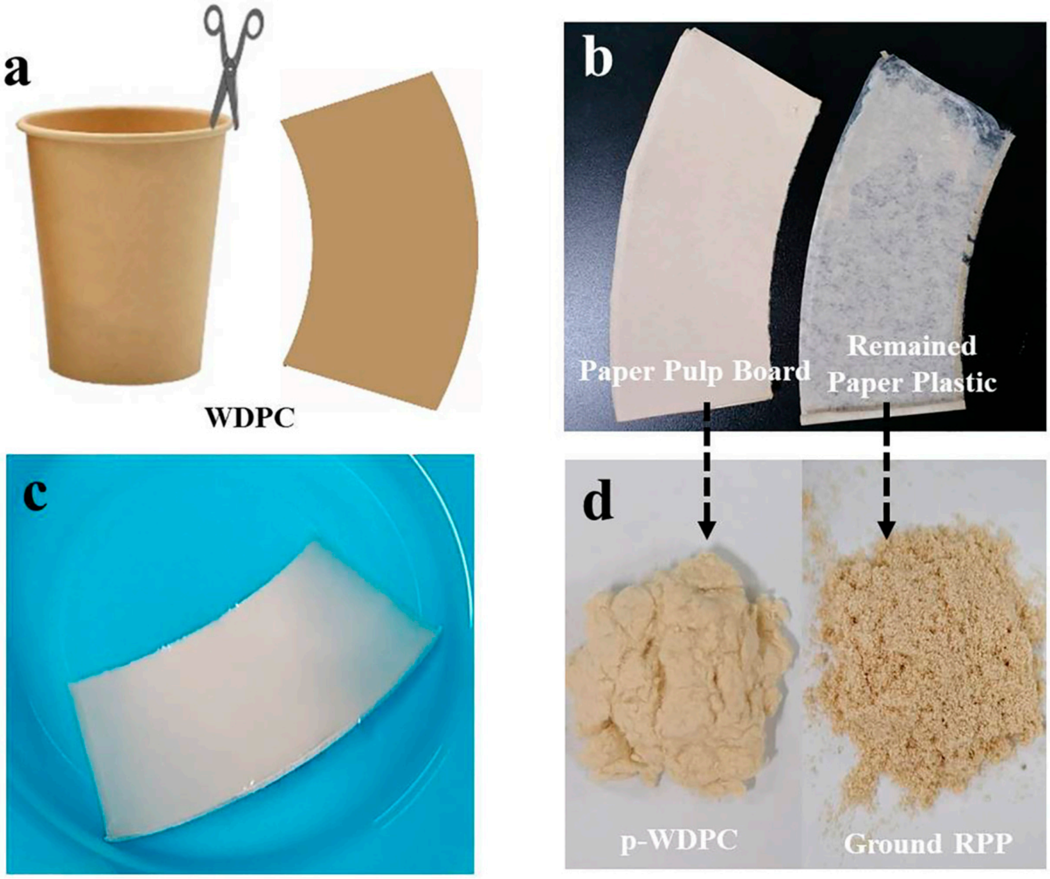

Figure S1. The photographs of (a) waste disposable paper cups (WDPC), (b) paper pulp board and remained paper plastic (RPP), (c) waste disposable paper cups soaked in deionized water and (d) shredded paper pulp board (p-WDPC) and ground RPP.

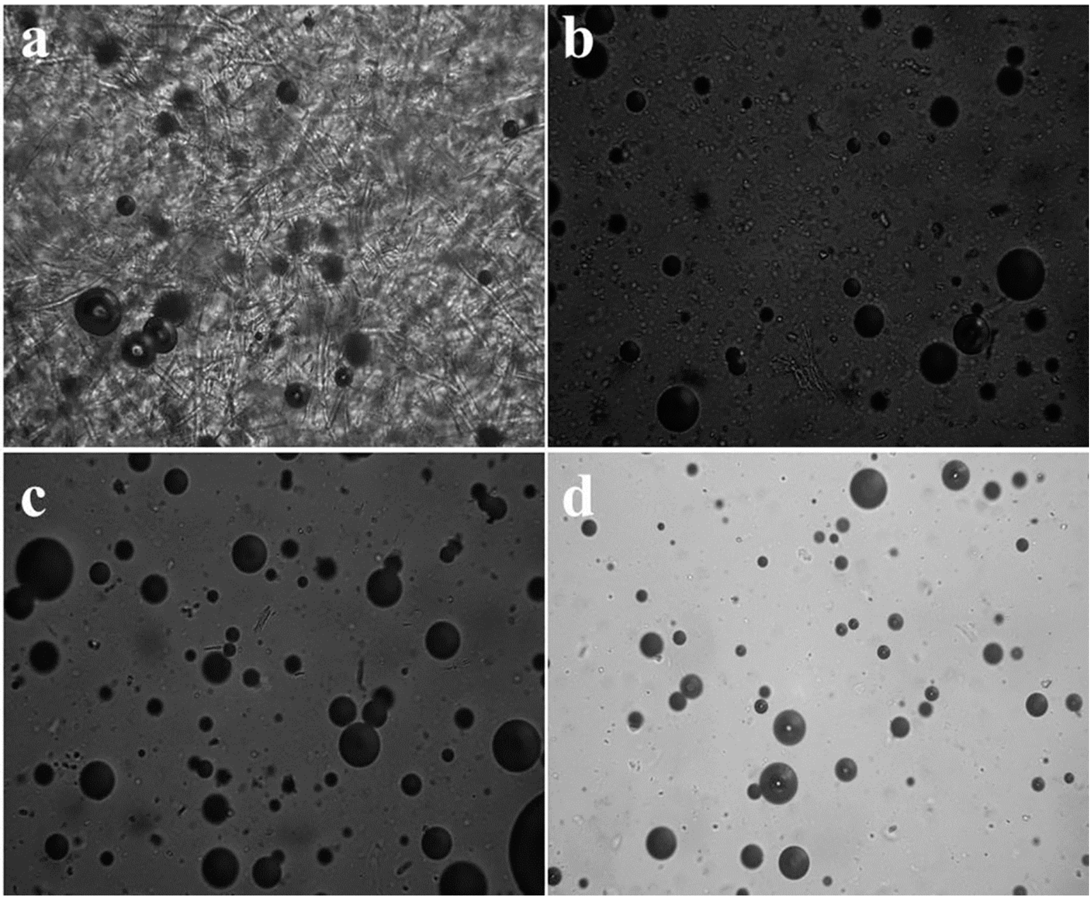

Figure S2. POM micrographs (10 × 10) of p-WDPC/AmimCl solution at 80 °C after 0 min(a), 30 min(b), 60 min(c) and 180 min(d).

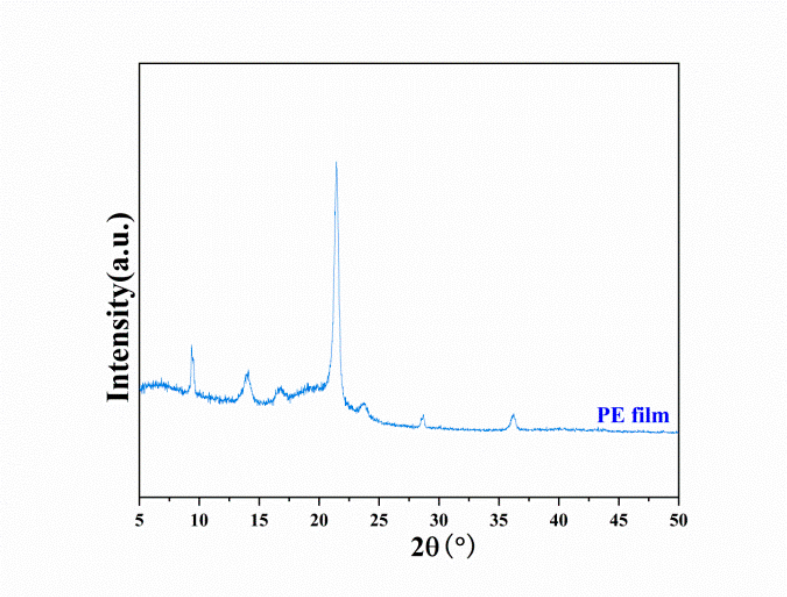

Figure S3. The XRD profile of commercial PE film.

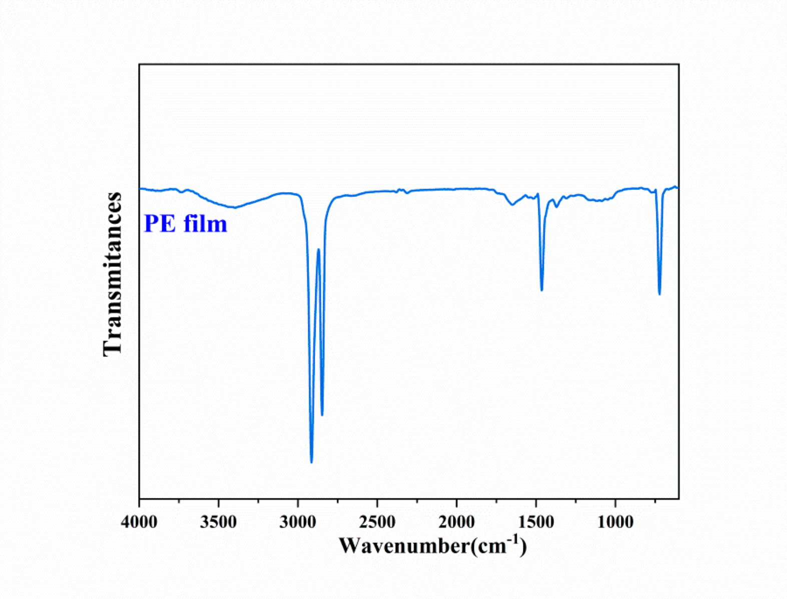

Figure S4. The FTIR spectrum of commercial PE film.

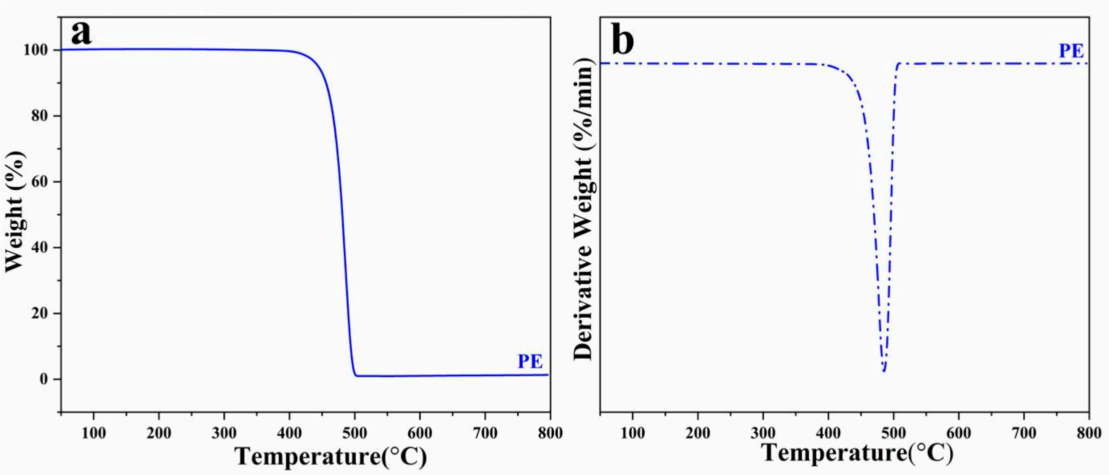

Figure S5. The (a) TG and (b) DTG curve of commercial PE film.

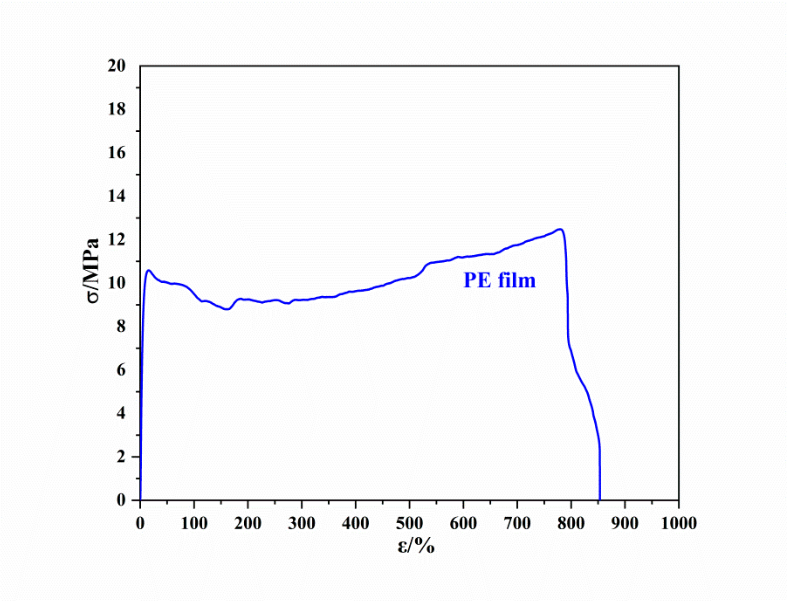

Figure S6. The stress-strain curve of commercial PE film.

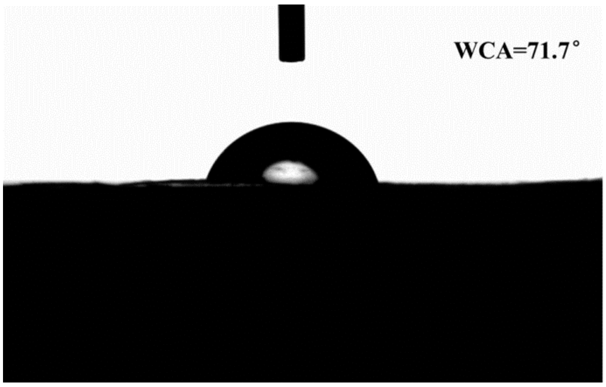

Figure S7. The water contact angle (WCA) of commercial PE film.
